# Single-center outcome analysis of 46 fetuses with megacystis after intrauterine vesico-amniotic shunting with the Somatex®intrauterine shunt

**DOI:** 10.1007/s00404-022-06905-6

**Published:** 2023-01-05

**Authors:** I. Gottschalk, C. Berg, T. Menzel, J. S. Abel, A. Kribs, M. Dübbers, J. Kohaut, L. T. Weber, C. Taylan, S. Habbig, M. C. Liebau, T. M. Boemers, E. C. Weber

**Affiliations:** 1 Division of Prenatal Medicine, Fetal Surgery and Gynecological Ultrasound, Department of Obstetrics and Gynecology, University Hospital Cologne and Faculty of Medicine, University of Cologne, Cologne, Germany; 2 Department of Neonatology, University Hospital of Cologne, Cologne, Germany; 3 Division of Pediatric Surgery, University Hospital of Cologne, Cologne, Germany; 4 Department of Pediatrics, University Hospital Cologne and Faculty of Medicine, University of Cologne, Cologne, Germany; 5 Department of Pediatric Surgery and Urology, Children´S Academic Hospital Amsterdamer Cologne, Cologne, Germany

**Keywords:** Lower urinary tract obstruction, Megacystis, Somatex®, Shunting, Prenatal, Ultrasound

## Abstract

**Objectives:**

To assess the spectrum of underlying pathologies, the intrauterine course and postnatal outcome of 46 fetuses with megacystis that underwent intrauterine vesico-amniotic shunting (VAS) with the Somatex® shunt in a single center.

**Methods:**

Retrospective analysis of 46 fetuses with megacystis that underwent VAS either up to 14 + 0 weeks (early VAS), between 14 + 1 and 17 + 0 weeks (intermediate VAS) or after 17 + 0 weeks of gestation (late VAS) in a single tertiary referral center. Intrauterine course, underlying pathology and postnatal outcome were assessed and correlated with the underlying pathology and gestational age at first VAS.

**Results:**

46 fetuses underwent VAS, 41 (89%) were male and 5 (11%) were female. 28 (61%) fetuses had isolated and 18 (39%) had complex megacystis with either aneuploidy (*n* = 1), anorectal malformations (*n* = 6), cloacal malformations (*n* = 3), congenital anomalies overlapping with VACTER association (*n* = 6) or Megacystis–Microcolon Intestinal–Hypoperistalsis Syndrome (MMIHS) (*n* = 2). The sonographic ‘keyhole sign’ significantly predicted isolated megacystis (*p* < 0.001). 7 pregnancies were terminated, 4 babies died in the neonatal period, 1 baby died at the age of 2.5 months and 34 (74%) infants survived until last follow-up. After exclusion of the terminated pregnancies, intention-to-treat survival rate was 87%. Mean follow-up period was 24 months (range 1–72). The underlying pathology was highly variable and included posterior urethral valve (46%), hypoplastic or atretic urethra (35%), MMIHS or prune belly syndrome (10%) and primary vesico-ureteral reflux (2%). In 7% no pathology could be detected postnatally. No sonographic marker was identified to predict the underlying pathology prenatally. 14 fetuses underwent early, 24 intermediate and 8 late VAS. In the early VAS subgroup, amnion infusion prior to VAS was significantly less often necessary (7%), shunt complications were significantly less common (29%) and immediate kidney replacement therapy postnatally became less often necessary (0%). In contrast, preterm delivery ≤ 32 + 0 weeks was more common (30%) and survival rate was lower (70%) after early VAS compared to intermediate or late VAS. Overall, 90% of liveborn babies had sufficient kidney function without need for kidney replacement therapy until last follow-up, and 95% had sufficient pulmonary function without need for mechanical respiratory support. 18% of babies with complex megacystis suffered from additional health restrictions due to their major concomitant malformations.

**Conclusions:**

Our data suggest that VAS is feasible from the first trimester onward. Early intervention has the potential to preserve neonatal kidney function in the majority of cases and enables neonatal survival in up to 87% of cases. Despite successful fetal intervention, parents should be aware of the potential of mid- or long-term kidney failure and of additional health impairments due to concomitant extra-renal anomalies that cannot be excluded at time of intervention.

## What does this study add to the clinical work

**Table:** 

Fetal megacystis comprises a heterogeneous group of conditions with high mortality if untreated prenatally. The underlying pathology of megacystis is highly variable, but prenatal differentiation is not possible. As prenatal vesico-amniotic shunting (VAS) is feasible from the first trimester onward, it should be performed as early as possible, because it has the potential to preserve neonatal kidney function in the majority of cases and enables neonatal survival in up to 87% of cases. But despite successful fetal intervention, parents should be aware of the potential risk of mid- or long-term kidney failure as the long-term effect of VAS on kidney function is unknown. In addition, the prevalence of concomitant severe extra-renal anomalies is high. Therefore, serial ultrasound examinations are mandatory after fetal intervention, because a significant proportion of these anomalies cannot be detected at the time of intrauterine intervention.	

## Introduction

Fetal megacystis comprises a heterogeneous group of conditions. The underlying pathology is highly variable ranging from isolated obstructive megacystis due to posterior urethral valves (PUV) to complex cloacal anomalies (CA) or non-obstructive neurogenic megacystis in Megacystis–Microcolon Intestinal–Hypoperistalsis Syndrome (MMIHS) [1]. Regardless of the underlying pathology, persistent megacystis due to restricted urinary bladder drainage may lead to pediatric kidney failure with high mortality, if untreated prenatally [2–8].

As postnatal therapy of megacystis is frequently too late to rescue kidney and respiratory function, several antenatal treatment options have been developed to enable continuous urinary drainage of megacystis into the amniotic cavity, including serial vesicocentesis [9–11], vesico-amniotic shunting (VAS) [12–26] or cystoscopic laser valve ablation [21, 27, 28]. All previously published series on antenatal interventions including our own previous studies [20, 24, 25] showed an increase in perinatal survival with the best renal and respiratory outcome after VAS. Indeed, data on intervention-related complications, intrauterine demise, preterm rupture of membrane and preterm delivery varied considerably between the published series, as the numbers of treated fetuses were small and VAS was performed by different operators in different centers at varying gestational ages with different shunt systems. In addition, the ideal gestational age for VAS is still unclear, as VAS before 17 weeks has been deemed unsuitable due to an assumed increased complication rate. However, in a previous comparative study we could show that VAS with the Somatex®shunt can already be performed safely before 17 + 0 weeks and survival rates as well as kidney und pulmonary function improved with the earlier gestational age at intervention [25]. Regardless of any intrauterine intervention, the outcome of megacystis remains difficult to predict due to the heterogenic underlying pathology and the high prevalence of complex concomitant urinary, anorectal and genital anomalies [29]. It has been demonstrated that the severity of these concomitant anomalies is the most powerful determinant of perinatal survival and morbidity [30].

The aim of this single-center study was to assess the outcome of a large cohort of fetuses with megacystis that underwent VAS exclusively with the Somatex®shunt at different gestational ages. The underlying pathologies and additional anomalies were assessed, the intrauterine course and all shunt-related complications are described and correlated with survival rate and postnatal morbidity. In addition, we looked for early sonographic predictors to prenatally identify the underlying pathology and to differentiate between obstructive and non-obstructive as well as isolated and complex megacystis, as this may have a significant impact on perinatal mortality and postnatal morbidity.

## Methods

We conducted a retrospective cohort study in a large tertiary referral center in Cologne, Germany, and included all cases of fetal megacystis that underwent intrauterine VAS with the Somatex® shunt with known outcome over a period of 6 years (2017–2022).

Megacystis in the first trimester was defined as bladder diameter ≥ 15 mm [31] and in the second and third trimester as a permanently enlarged bladder without evidence of adequate emptying in combination with reduced amniotic fluid volume. Hydronephrosis was defined as pelvic anterior–posterior diameter ≥ 4 mm in the first and ≥ 7 mm in the second trimester. All fetuses underwent several detailed anomaly scans, and karyotyping was offered to all parents.

Regarding the high prevalence of associated anomalies, we classified megacystis either as isolated or complex megacystis.

Isolated megacystis without major concomitant anomalies included megacystis due to

- Posterior urethral valve (PUV),
- Urethral stenosis, hypoplasia or atresia,
- Primary vesico-ureteral reflux (VUR) or
- Prune Belly syndrome (PBS).

Complex megacystis included megacystis with either concomitant

- Chromosomal anomalies,
- Anorectal malformations (ARM) with additional developmental anomalies of the distal anus, rectum or genitourinary tract or cloacal malformations,
- Multiple congenital abNormalities (MCA) overlapping with those of VACTERL association or
- Neurogenic bladder in Megacystis–Mikrocolon Intestinal Hypoperistalsis-Syndrome (MMIHS).

Due to the overlapping use of the terms Cloacal Malformation, Cloacal Dysgenesis Sequence and Urorectal Septum Malformation Sequence in literature, we defined cloacal malformation in our cohort as absence of anal and genitourinary orifices and confluence of rectum, vagina and/or bladder in the urogenital sinus (as defined by Wheeler et Weaver [32]).

After first diagnosis, all fetuses underwent VAS with the Somatex® Intrauterine shunt (Somatex Medical Technologies, Berlin, Germany) immediately or, if fetal position was unfavorable for immediate VAS, within the next 24 h. VAS was offered from 11 + 0 weeks of gestation onward and was exclusively performed by one of two experienced fetal operators (either C.B. or I.G.) using a technique as previously described by Strizek et al. [24, 25]. Genetic testing was offered to all patients, but was not mandatory prior to intrauterine intervention. If genetic testing was not yet done but desired by the patients, karyotyping was performed during VAS, as the design of the Somatex® Intrauterine shunt allows to acquire amniotics fluid through the same cannula after shunt deployment. If amniotic fluid aspiration during VAS failed, chorionic villus sampling or amniocentesis was performed depending on the gestational age.

If deemed necessary, amniotic fluid was infused prior to VAS using an 18gauge spinal needle and 5% glucose solution. After VAS, all fetuses underwent detailed two weekly ultrasound anomaly scans to assess the fetal anatomy and the shunt position and function. In cases of shunt dislocation or migration, VAS was repeated as soon as possible.

All pediatric and surgical medical files of postnatal ultrasound, micturating cystourethrography or surgery were reviewed until final follow-up to establish the final diagnosis and to assess the underlying pathology, all neonatal complications, the postnatal surgical interventions and therapy. Kidney function within the first days of life was either assessed by local age-dependent reference values for creatinine, ß2-microglobulin and urea or by description of renal function in external pediatric records and charts. All follow-up examinations were either performed in our institutional departments of pediatrics, pediatric surgery and urology or in the participating local centers for pediatrics in standardized procedures.

Three patients of out cohort that underwent VAS before 2018 were already included in a previous feasibility study [24] and their outcome has already been published in another previous publication [25].

In a subgroup analysis, the outcome was correlated with regard to gestational age at first shunting. First VAS up to 14 + 0 weeks was classified as early VAS group, first VAS between 14 + 1 and 17 + 0 weeks as intermediate VAS group and VAS after 17 + 1 week gestation as late VAS group.

Statistical analysis was performed using the Statistical Package for Social Sciences (SPSS 22.0, SPSS Inc., Chicago, Ill., USA) statistical software. A *p* value of < 0.05 was considered significant. This retrospective study was approved by the local ethical committee of human research (No 20–1517).

## Results

During the 6-year study period, 46 cases of fetal megacystis were referred to our center, including 1 dichorionic twin and 45 singleton pregnancies. Among the 46 affected fetuses, 41 (89.1%) were male and 5 (10.9%) were female. At first ultrasound all fetuses had megacystis and bilateral hydronephrosis, 39 (84.8%) fetuses had an additional ‘keyhole sign’.

### Characteristics of the cohort

Twenty-eight (60.9%) fetuses had isolated megacystis without any major additional anomalies and 18 (39.1%) had complex megacystis with major additional anomalies (Table 1). With the exception of the hymenal atresia that was postnatally diagnosed all additional anomalies were correctly described prenatally and confirmed postnatally. The three most common major additional anomalies were imperforated anus in 26%, followed by recto-genito-urinary fistulas and genital malformations in 12% each (Table 2). Although a proportion of these additional anomalies escaped detection at first trimester ultrasound, prenatal diagnosis was achieved during subsequent ultrasound examinations (with the exception of hymenal atresia).

**Table 1:** Antenatal characteristics, outcome and postnatal findings in 46 cases with megacystis

	GA @ 1st diagnosis	Sex	Isolated/complex megacystis	Keyhole-sign	Additional anomalies @ 1st diagnosis	AFI @ 1st diagnosis	GA @ 1st shunting	Amnion infusion before shunting	Shunt complications	Additional shunting	Underlying cause postnatally	Postnatal diagnosis	Additional extra-renal anomalies postnatally confirmed	Additional renal anomalies postnatally confirmed	gestational age @ birth	Outcome	Impairment postnatally
1	13 + 0	Female	Complex	Yes	Pes equinovarus	Normal	13 + 0	–	–	–	–	MCA	Lumbo–sacral myelomeningocele + esophageal atresia	–	–	TOP	–
2	11 + 4	Male	Complex	Yes	Nasal bone aplasia, enterolithiasis	Normal	12 + 5	–	–	–	–	MCA	Hydrocephaly + thoraco-lumbal scoliosis	–	–	TOP	–
3	13 + 1	Male	Complex	Yes	–	Normal	13 + 1	–	–	–	–	Chrosmosomal	Trisomy 18	–	–	TOP	–
4	12 + 1	Male	Complex	Yes	–	Normal	13 + 6	–	–	–	–	ARM	Imperforated anus	MCDKD, contralateral renal dysplasia	–	TOP	–
5	12 + 6	Female	Complex	No	–	Normal	12 + 6	–	–	–	Urethral atresia	Cloacal malformation	Imperforate anus + vesico-intestinal fistula + indifferential genitalia	Renal agenesis,contralateral renal dysplasia	30 (PROM + cord prolapse)	NND	Palliative care
6	13 + 6	Male	Complex	Yes	Dichorionic twins	Oligo	13 + 6	–	Iatrogenic shunt displacement	Vesico-amn	Urethral atresia	ARM	Imperforate anus + clubfeet + pulmonary hypoplasia	–	24 (amniotic sac prolapse + preterm labor)	NND	Extreme prematurity, palliative care
7	13 + 6	Male	Isolated	Yes	–	Normal	13 + 6	–	–	–	PUV		–	Bilateral double kidney, unilateral urinoma	33 (PROM)	CHD	Lethal lung hypoplasia
8	13 + 4	Male	Complex	Yes	Cord cyst	Normal	13 + 4	–	–	–	Urethral hypoplasia	ARM	Imperforated anus + rectoprostatic fistula	–	39	Survivor	–
9	12 + 6	Male	Isolated	Yes	–	Normal	12 + 6	–	–	–	No reason found		–	Severe bilateral megaureters	39	Survivor	–
10	12 + 6	Male	Complex	No	Cord cysts,hyper-echogenic intestines, enterolithiasis	Oligo	13 + 3	Yes	–	–	Urethral atresia	ARM	Imperforated anus + rectourethral fistula	–	40	Survivor	–
11	13 + 6	Male	Isolated	Yes	Bilateral club feet	Oligo	13 + 6	–	Spontaneous dislocation @ 30 weeks	Abd-amn	PUV		Cryptorchism	Unilateral double kidney	32 (PROM)	Survivor	–
12	13 + 5	Male	Isolated	Yes	–	Normal	13 + 5	–	Spontaneous dislocation @ 17 weeks	Abd-amn	Urethral stenosis, urethral hypoplasia		Cryptorchism + intestinal perforation due to intra-abdominally dislocated shunt	Unilateral double kidney	38	Survivor	–
13	14 + 0	Male	Isolated	No	–	Oligo	14 + 0	–	–	–	No reason found		–	Unilateral urinoma	40	Survivor	–
14	14 + 0	Male	Isolated	Yes	–	Normal	14 + 0	–	–	–	PUV, urethral hypoplasia		–	Unilateral urinoma	39	Survivor	–
15	14 + 3	Male	Complex	Yes	Atypically shaped megacystis with ventral diverticle	Normal	14 + 5	-	–	–	–	MCA/VATER	Imperforate anus + duodenal atresia	Unilateral MCDKD	–	TOP	–
16	14 + 2	Male	Complex	Yes	Cervical MMC	Oligo	14 + 3	Yes	–	–	–	MCA/VACTER	Myelomeningocele + severe fetal growth restriction (FGR) + Coarctatio aortae	–	–	TOP	–
17	13 + 3	Female	Complex	Yes	–	Normal	14 + 3	–	–	–	–	MCA/VATER	Spinal dysraphia + imperforated anus	Horseshoe kidney	–	TOP	–
18	14 + 3	Female	Complex	Yes	–	anhydr	14 + 3	Yes	–	–	Urethral atresia	Cloacal malformation	Hymenal atresia (postnatally diagnosed) + imperforated anus + rectouterine fistula	Bilateral renal hypoplasia	36	NND	Lethal respiratory insufficiency
19	14 + 3	Male	Complex	Yes	Atypically shaped megacystis	Oligo	14 + 3	Yes	–	–	Urethral atresia, urethral hypoplasia	ARM	Imperforate anus + scrotum bifidum + penoscrotal transposition	Unilateral renal hypoplasia	40	NND	End-stage renal failure, uremic pericarditis
20	14 + 5	Male	Isolated	Yes	–	Oligo	14 + 5	Yes	–	–	Prune belly		Mild prune belly + severe megaureters + VUR III°	–	38	Survivor	Mildly impaired by recurrent infections and prune belly
21	14 + 3	Female	Complex	No	SUA	Oligo	14 + 3	–	–	–	Urethral atresia	Cloacal malformation	Hymenal atresia + imperforate anus + indifferent genialia + scoliosis + cor triatriatum + aplasia cutis congenita right thigh	Horseshoe kidney	40	Survivor	Moderately impaired by stoma
22	14 + 3	Male	Isolated	Yes	ARSA	Oligo	14 + 3	–	–	–	PUV		Urethral duplication + VUR left	Unilateral renal hypoplasia	40	Survivor	–
23	14 + 2	Male	Complex	No	Intestinal dilatation, enterolithiasis	Normal	14 + 2	–	–	–	Urethral hypoplasia	ARM	Imperforated anus + rectovesical fistula + VUR	Unilateral renal hypoplasia	39	Survivor	Mildly impaired by stoma
24	13 + 1	Male	Isolated	Yes	–	Normal	14 + 1	–	–	–	VUR		–	–	38	Survivor	–
25	14 + 2	Male	Complex	Yes	Cord cyst, megalourethra	Normal	14 + 2	–	–	–	Urethral hypoplasia	ARM	Imperforated anus + recto-vesical fistula + clumsy penis + scrotum bifidum + cryptorchism	Unilateral renal hypoplasia	40	Survivor	–
26	14 + 1	Male	Complex	No	Inestinal dilatation	Normal	14 + 1	–	Spontaneous dislocation @ 31 weeks	Abd-amn	Neurogenic	MMIHS	Congenital mikrocolon + intestinal malrotation + clubfeet	Bilateral hydronephrosis + megaureters	31 (AIS)	Survivor	Mildly impaired by stoma
27	15 + 1	Male	Complex	No	–	Normal	15 + 1	–	–	–	Neurogenic	MMIHS	Congenital microcolon	Bilateral hydronephrosis + megaureters	37	Survivor	Severely impaired by parenteral nutrition
28	15 + 4	Male	Isolated	Yes	–	Oligo	15 + 4	–	–	–	PUV		Mild pulmonary hypoplasia + mild muscular hypotonia	–	38	Survivor	–
29	15 + 0	Male	Isolated	Yes	–	Oligo	15 + 1	–	–	–	PUV		Cryptorchism	–	37	Survivor	Asymptomatic mild chronic renal insufficiency
30	15 + 2	Male	Isolated	Yes	–	Oligo	15 + 2	–	Spontaneous dislocation @ 27 weeks	Abd-amn	PUV		–	–	31 (PROM)	Survivor	–
31	15 + 1	Male	Isolated	Yes	–	Oligo	15 + 1	Yes	–	–	Prune belly		Prune belly syndrome + cryptorchism	–	32 (AIS)	Survivor	Mildly impaired due to aplasia of abdominal muscles
32	16 + 2	Male	Isolated	Yes	–	Oligo	16 + 1	–	–	–	PUV		–	–	38	Survivor	–
33	16 + 4	Male	Isolated	Yes	PA + VSD + MAPCAs	Normal	16 + 4	–	–	–	PUV		Pulmonary valve atresia with VSD and MAPCAs	–	39	Survivor	–
34	16 + 5	Male	Isolated	Yes	–	Normal	16 + 5	–	Spontaneous dislocation @ 28 and 31 weeks	Abd-amn	PUV, urethral hypoplasia		–	–	36	Survivor	–
35	16 + 3	Male	Isolated	Yes	–	Oliog	16 + 3	Yes	–	–	Urethral hypoplasia		–	–	39	Survivor	–
36	16 + 6	Male	Isolated	Yes	–	Oligo	16 + 6	–	Spontaneous dislocation @ 29 weeks	Abd-amn	PUV		–	–	37	Survivor	–
37	16 + 2	Male	Isolated	Yes	–	Normal	16 + 2	–	–	–	No reason found		Pulmonary hypoplasia	Bilateral cystic dysplastic kidneys	40	Survivor	Requiring dialysis
38	15 + 6	Male	Isolated	Yes	–	Oligo	15 + 6	Yes	–	–	PUV		Unilateral VUR	Unilateral cystic dysplastic kidney	34 (PROM)	Survivor	–
39	18 + 2	Male	Isolated	Yes	Unilateral renal agenesis	Oligo	18 + 2	Yes	Spontaneous dislocation @ 27 weeks	Abd-amn	PUV		Aplasia cutis right thigh	Unilateral renal agenesis	36	Survivor	–
40	18 + 1	Male	Isolated	Yes	–	Oligo	18 + 1	–	–	–	PUV		–	Bilateral hypoplastic kidneys	37	Survivor	–
41	19 + 4	Male	Isolated	Yes	–	Oligo	18 + 2	Yes	–	–	PUV		Urachus diverticulum + mild pulmonary hypoplasia	Cystic dysplastic kidney, contralateral dysplastic double kidney	36	Survivor	Required kidney-transplantation
42	19 + 6	Male	Isolated	Yes	–	Anhydr	18 + 3	Yes	Iatrogenic shunt displacement	Vesico-amn	Long-distance urethral hypoplasia		Cryptorchism	Cystic dysplastic kidney, contralateral urinoma	36	Survivor	–
43	20 + 4	Male	Isolated	Yes	–	Oligo	21 + 4	–	Spontaneous dislocation @ 30 weeks	Abd-amn	PUV		VUR IV° + omental prolaps (iatrogenic gastroschisis)	Unilateral urinoma	35	Survivor	Asymptomatic mild chronic renal insufficiency
44	24 + 0	Male	Isolated	Yes	–	Oligo	24 + 0	Yes	Spontaneous dislocation @ 28 weeks	Abd-amn	PUV		–	–	39	Survivor	–
45	24 + 5	Male	Isolated	Yes	–	Oligo	24 + 5	Yes	–	–	PUV		–	–	29 (PROM)	Survivor	–
46	25 + 0	Male	Isolated	Yes	–	Anhydr	25 + 2	Yes	Iatrogenic shunt displacement	Vesico-amn	Hypoplastic urethra		–	Bilateral cystic dysplastic kidneys	37	Survivor	Requiring dialysis

**Table 2:** Type and prevalence of additional anomalies in 18 cases with complex megacystis

Type of additional anomalies	Prevalence *n* (%)
Imperforate anus	12 (26)
Fistulas (recto-vesical, recto-prostatic, recto-urethral, recto-uterine)	6 (12)
Genital malformations (ambiguous genitalia, scrotum bifidum, clumsy penis cryptorchism)	6 (12)
Neural tube defects (myelomeningocele, spinal dysgraphia)	3 (6)
Cardiac anomalies	3 (6)
Esophageal/duodental atresia	2 (4)
vertebral scoliosis	2 (4)
Clubfeet (without neural tube defects)	2 (4)
Hymenal atresia (*)	2 (4)
Congenital megacystis–microcolon intestinal–hypoperistalsis syndrome	2 (4)
Aplasia cutis congenita	2 (4)
Hydrocephaly (without neural tube defect)	1 (2)
Trisomy 18	1 (2)
Severe fetal growth restriction (FGR)	1 (2)

*Postnatally diagnosed

Among the 5 female fetuses all had complex megacystis with additional cloacal malformation in 3 cases and VACTERL association in 2 fetuses. Two of the five pregnancies were terminated, 2 babies died in the neonatal period, and 1 girl survived. She was moderately impaired by her stoma at last follow-up at an age of 4 years. Therefore, female sex was a significant predictor of severe and complex megacystis with adverse outcome.

Prenatal genetic testing prior to or during VAS was performed in 24 (52.2%) cases. In the remaining 22 (47.8%) patients, genetic testing was carried out postnatally. All but one case had a normal karyotype (97.8%). In the only case with abnormal karyotype, the initial genetic testing during VAS failed due to insufficient cell growth, making an amniocentesis inevitable 2 weeks later. Karyotyping then revealed a trisomy 18. Consequently, this pregnancy was terminated.

### VAS

Early VAS was performed in 14 (30.4%) cases, intermediate VAS in 24 (52.2%) cases and late VAS in 8 (17.4%) cases (Table 3). Technically successful first VAS was achieved in 43 (93.5%) cases, whereas in three (6.5%) cases the initial shunt was positioned incorrectly into the fetal abdomen and a second shunt had to be placed within the next day. Amniotic fluid index at first diagnosis was significantly reduced in 26 (56.5%) cases and amnion infusion prior to VAS was warranted in 14 (30.4%) cases.

**Table 3:** Characteristics of early, intermediate and late VAS subgroups

	Early VAS ≤ 14 + 0 weeks	Intermediate VAS 14 + 1–17 + 0 weeks	Late VAS > 17 + 0 weeks	All	Significance (*p* < 0.05)
Number of fetuses (*n*)	14	24	8	46	
Prevalence of isolated megacystis	6/14 (42.9%)	14/24 (58.3%)	8/8 (100%)	28/46 (60.9%)	*p* = 0.029*
Amnion infusion prior to VAS	1/14 (7.1%)	7/24 (29.2%)	6/8 (75%)	14 /46 (30.4%)	*p* = 0.004*
1st VAS (weeks of gestation)	13 + 1 (range, 11 + 4–14 + 0)	15 + 1 (range, 14 + 1–16 + 6)	21 + 6 (range, 18 + 1–25 + 2)	15 + 6 (range, 11 + 4–25 + 2)	
Shunt complications	4/14 (28.6%)	5/24 (20.8%)	6/8 (75%)	15/46 (32.6%)	*p* = 0.015*
TOP	4/14 (33.3%)	3/24 (12.5%)	0/8 (0%)	7/46 (15.2%)	
Preterm delivery ≤ 32 weeks	3/10 (30%)	3/21 (14.3%)	1/8 (12.5%)	7/39 (17.9%)	*p* = 0.512
Gestational age @ delivery	31 + 5 (range, 23 + 1–40 + 6)	36 + 5 (range, 30 + 5–40 + 0)	35 + 1 (range, 28 + 0–38 + 6)		
Intention to treat survival	7/10 (70%)	19/21 (90.5%)	8/8 (100%)	34/39 (87.2%)	*p* = 0.134
Restricted renal function @birth	0/10 (0%)	3/21 (14.3%)	3/8 (37.5%)	6/39 (15.4%)	*p* = 0.089
Restricted pulmonary function @birth	1/10 (10%)	1/21 (4.8%)	0/8 (0%)	2/39 (5.1%)	*p* = 0.629
Follow-up (months)	18 (range, 2–68)	27 (range, 1–72)	36 (range, 2–72)	24 (range, 1–72)	

*Statistically significant, the early VAS group was compared to the intermediate and late VAS groups

Further shunt complications such as spontaneous dislocation or migration of the shunt into the fetal abdomen in the further course of pregnancy occurred in another 10 (21.7%) cases, at a median gestational age of 28 weeks (range, 17–31). In these cases, an additional peritoneo-amniotic shunt was placed in the fetal abdominal wall to drain the urinary ascites. Fetal injuries due to the shunt itself occurred in another 2 (4.3%) cases and included intestinal perforation by the dislocated shunt in one newborn and a small abdominal wall defect with omental prolapse at the shunt insertion site in another newborn baby.

Therefore, the likelihood of an unsuccessful first VAS in our cohort was 6.5% and the necessity of a second and third VAS due to spontaneous shunt dislocation was 19.5% and 2.2%, respectively. No spontaneous intrauterine demise occurred.

Overall, preterm delivery prior to 32 + 0 weeks of gestation due to preterm rupture of membrane (PROM), amniotic sac-prolapse, amniotic infection syndrome or preterm labor occurred in 17.9% and was more common after early VAS (30%) (*p* = 0.512), although these numbers did not reach significance. Median gestational age at delivery was 35 + 1 weeks of gestation (range, 30 + 5–40 + 0). Median follow-up was 72 months (range, 2–72).

### Mortality, survival rate and postnatal morbidity

**Fig. 1:**
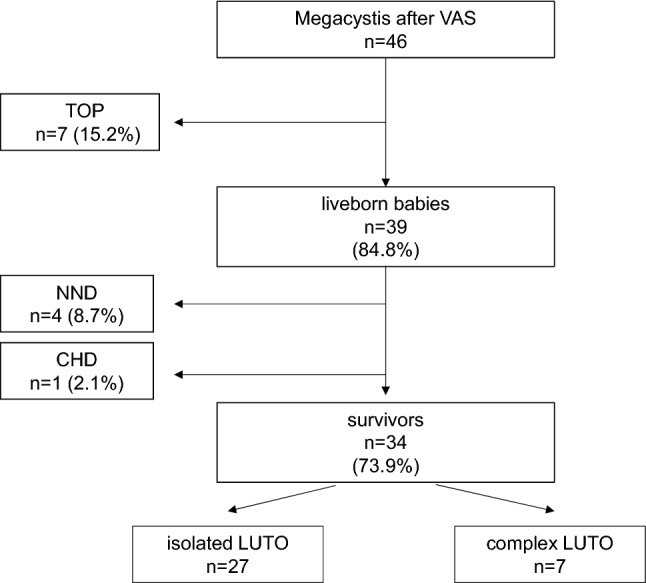
Outcome of the 46 fetuses with megacystis after VAS

Seven (15.2%) pregnancies were terminated (TOP), all had complex megacystis (Table 1, Fig. 1). Four (8.7%) liveborn babies died in the neonatal period (NND), all had complex megacystis. Two of them were born at 23 and 29 weeks of gestation and died after palliative care within the first 10 h of life, one died of lung hypoplasia after 8 days of life and another one after 9 days due to uremic pericarditis. Another male baby died of sequelae of lung hypoplasia at the age of 2.5 months. He had isolated megacystis and underwent early VAS.

Thirty-four (73.9%) babies were alive at last follow-up. Median follow-up was 24 months (range, 1–72 months). After exclusion of TOP, the mortality rate in our cohort was 12.8% with an overall survival of 87.2%. However, there was a significantly higher survival amongst fetuses with isolated (96.4%) versus complex megacystis (63.6%) (*p* = 0.025).

Among the 39 liveborn babies, 33 (84.6%) babies had sufficient kidney function without need for immediate dialysis after birth as well as at last follow-up at a median age of 2 years of age (range 1–6 years). Two (5.1%) babies were born with asymptomatic mild chronic renal insufficiency without need of dialysis at last follow-up at a median age of 3.6 years, and 4 (10.3%) babies were born with kidney failure. Among them, one required kidney transplantation after his first year of life with well-functioning transplant at last follow-up 2 years later, and the remaining three were on chronic dialysis at a median age of 1.5 years of life. Kidney function was better after early VAS as all babies with impaired kidney function had either intermediate or late VAS. However, these findings did not reach significance (*p* = 0.089). Despite successful VAS, subsequent prenatal sonographies revealed significant alterations of the kidneys or the urinary tract in nearly all cases that could also be confirmed postnatally. Significant hydronephrosis or megaureters was diagnosed in 14 cases, cystic–dysplastic kidneys in 7, urinoma in 5, significant renal hypoplasia in 4 and unilateral renal agenesis in 3 cases.

Pulmonary size and function at birth was adequate in 37 (94.9%) of the 39 liveborn without need for mechanical respiratory support. Two (5.1%) babies had lung hypoplasia and fatal respiratory insufficiency. Pulmonary size and function did not differ between the subgroups of early, intermediate or late VAS.

With regard to the overall health status, 6 (17.6%) of the 34 survivors had additional health restrictions as a consequence of their concomitant extra-renal anomalies, either by their stoma due to underlying imperforate anus (*n* = 3), by their hypoplastic abdominal muscles due to the underlying prune belly syndrome (*n* = 2) or by parenteral nutrition due to an underlying MMIHS (*n* = 1). Accordingly, overall health status was better among survivors with isolated compared with complex megacystis (*p* = 0.116). Among survivors with isolated megacystis, 74.1% were healthy without any renal, pulmonary or other limitations, compared with only 42.9% survivors with complex megacystis. Due to the small number of survivors with complex megacystis the result did not reach statistical significance (*p* = 0.116).

### Underlying pathology of megacystis

Most common underlying pathologies of megacystis among the 39 liveborn babies with known outcome were PUV in 18 (46.2%) cases, followed by hypoplastic or stenotic urethra in 7 (20.6%) and atretic urethra in 6 (15.4%) cases, neurogenic bladder due to MMIHS or Prune belly syndrome in 2 (5.1%) cases, each, and primary VUR due to insufficient flap-valves in 1 (2.6%) case (Table 1). In 3 (7.7%) cases no underlying pathology for megacystis could be detected postnatally, although all 3 newborns had significant additional urinary abnormalities including megaureters, urinoma or end-stage cystic dysplastic kidney disease.

### ‘Keyhole sign’

Among the 39 liveborn babies with known outcome, 32 (82.1%) had megacystis with ‘keyhole sign’ at time of first diagnosis and 7 (17.9%) had megacystis without ‘keyhole sign’ (Table 1). Among those with ‘keyhole sign’, 27 (84.4%) had isolated megacystis and 5 (15.6%) had complex megacystis with either additional anorectal (*n* = 4) or cloacal malformations (*n* = 1). Twenty-five (78.1%) of the 32 babies with ‘keyhole sign’ survived without severe postnatal impairment. In contrast, among the 7 babies without ‘keyhole sign’, only 1 (14.3%) had isolated and 6 (85.7%) had complex megacystis with either additional anorectal (*n* = 2), cloacal malformations (*n* = 2) or MMIHS (*n* = 2). Only 2 (28.6%) of the 7 babies without ‘keyhole sign’ survived without any impairment. Therefore, the presence of the ‘keyhole sign’ was significantly associated with isolated megacystis and consequently with an overall better outcome in our cohort (*p* < 0.001).

However, the ‘keyhole sign’ was no reliable marker to predict the underlying pathology or to differentiate between obstructive or non-obstructive megacystis (*p* = 0.137). Among the 32 babies with ‘keyhole sign’, 27 (84,4%) had obstructive megacystis due to PUV or hypoplastic or atretic urethra and 5 (15.6%) had non-obstructive megacystis. In contrast, among the 7 babies without ‘keyhole sign’ only 3 (42.9%) babies had non-obstructive and 4 (57.1%) had obstructive megacystis.

When these results are analyzed according to the gestational age at VAS, amnion infusion was significantly more often necessary prior to intermediate or late VAS than to early VAS (*p* = 0.004) and shunt complications occurred significantly more often after late VAS (*p* = 0.015), whereas preterm delivery ≤ 32 + 0 weeks occurred noticeably more often after early VAS (*p* = 0.512). Prevalence of isolated megacystis was significantly higher (*p* = 0.029) and survival rates noticeably higher in the intermediate or late VAS group (*p* = 0.134), but renal function was noticeably better after early VAS (*p* = 0.089) (Table 2).

## Discussion

We present the outcome of a large cohort of fetuses with megacystis treated by vesico-amniotic shunting (VAS) with the Somatex® Intrauterine Shunt by two experienced operators in a single center. Our data suggest that VAS is feasible from the first trimester onward and early intervention has the potential to preserve kidney function in the majority of cases.

Mild megacystis < 15 mm is often a transient finding in the first trimester, that spontaneously resolves during ongoing weeks of pregnancy [7, 31, 33, 34], whereas megacystis > 15 mm is strongly suggestive of significant restricted urinary bladder drainage and a spontaneous resolution is unlikely [1–5, 8]. Untreated, a persistent megacystis is associated with high mortality and severe postnatal morbidity caused by lung hypoplasia and impaired kidney function, regardless of the underlying pathology [2–6, 8, 16, 17, 35–39].

Several authors have evaluated prognostic factors to predict the risk of kidney failure [4–6, 8, 21, 33, 40–45] and different fetal therapies have been evaluated to enable continuous drainage into the amniotic cavity and to prevent renal damage and pulmonary hypoplasia [46]. In a recently published review, the ERKNet CAKUT-Obstructive Uropathy Work Group confirmed data from the randomized PLUTO trial [16, 17] and recommended VAS as the therapy of choice in selected cases with moderate and severe megacystis, ideally performed before 27 weeks of gestation [8]. In three previously published own series we also confirmed that VAS is a safe and effective intervention that can enable survival in up to 78% and preserves normal kidney function in up to 86% of the survivors [20, 24, 25].

The embryonic kidneys develop from the fourth week of gestation onward and nephrogenesis passes through 3 stages [47]. Any disturbance during this period may negatively influence the normal ureteric bud branching and metanephric mesenchymal differentiation. As the duration of urinary obstruction is crucial for the development of renal dysplasia [48], fetal intervention should be performed as soon as possible after first diagnosis of megacystis. However, so far, there are only very few studies on VAS before 17 weeks of gestation, because it has been considered to be associated with high complication rates [17, 26, 49]. However, in a previously published feasibility study we demonstrated that VAS using the Somatex® VAS system with its smaller introduction cannula is already feasible < 17 weeks without significantly more frequent shunt complications [24, 25]. In this study VAS was performed from the 12th week of gestation onward and kidney function could be preserved in all cases if VAS was performed ≤ 14 + 0 weeks of gestation. In addition, the amount of amniotic fluid is usually unchanged in first trimester megacystis; therefore, amnion infusion prior to VAS could be avoided in nearly all cases.

In previously published trials on VAS, the prevalence of shunt-related complications and survival rates differed significantly. This might be explained by poorly comparable and predominantly small cohorts in which different types of shunt systems were used in different centers with different local protocols and at different gestational ages (before or after 17 + 0 weeks of gestation) [12–26]. In contrast, our data are derived from a large single-center cohort, where exclusively, the Somatex® shunt was used. Overall survival in our cohort was 74% and noticeably better than those reported in literature, where 12 months survival rates of less than 44% are reported [16, 17, 22, 46].

In the literature, VAS-related complications occur in 40% of cases and include shunt dislocation or migration, shunt blockage, preterm rupture of membrane and preterm labor, abdominal wall herniation or intrauterine fetal death [8, 14–19, 23–25]. In our cohort shunt complications occurred in 22% of cases and were limited to shunt dislocation or migration and were even less common after early VAS compared with late VAS. There was no case of fetal loss. Intervention-related preterm delivery prior to 32 + 0 weeks due to preterm rupture of membrane or preterm labor occurred in 18% of cases. Therefore, we conclude that VAS is an effective treatment that ideally should be performed in the first trimester, as it shows the best results concerning kidney and pulmonary function, although it carries an 18% risk of preterm delivery.

More than one third of our cohort had complex megacystis (39%) with additional severe anomalies, including anorectal malformations with additional developmental anomalies of the distal anus, rectum or genitourinary tract, cloacal malformations or congenital malformations overlapping with those of VACTERL association. However, since kidney function can successfully be preserved by early VAS, the severeness of these additional anomalies influenced survival and postnatal morbidity more significantly than renal insufficiency. Consequently, survival in fetuses with complex megacystis was only 64% compared with 96% in isolated megacystis in our cohort. Unfortunately, a significant proportion of those additional anomalies, especially anorectal or cloacal malformations, escaped detection in the first trimester, when VAS was performed. This resulted in second trimester terminations of pregnancy in 15% of cases. In literature, about 50% of pregnancies were terminated after diagnosis of megacystis [2, 4–7, 16, 17, 31, 40, 50–52]. It may be assumed that the high termination rate in literature was caused mainly by parental fear of end-stage renal failure that may now be avoided by early VAS.

The etiology of megacystis is described to be highly variable causing obstructive or non-obstructive megacystis [2, 4–7, 16, 17, 31, 40, 51, 52]. Most commonly, obstructive megacystis in male fetuses is caused by isolated urethral anomalies such as posterior (or anterior) urethral valves in 57% of cases, urethral hypoplasia, stenosis or atresia or megalourethra in 7.4%. In these cases, the synonym lower urinary tract obstruction (LUTO) with megacystis can be used to correctly describe the situation. Most common non-obstructive megacystis are caused by vesico-ureteral reflux (VUR) that can mimic obstructive megacystis. Less commonly, but more complex are non-obstructive megacystis due to neuromuscular anomalies such as Prune Belly Syndrome (PBS) in 3.8%, Megacystis–Microcolon Intestinal–Hypoperistalsis Syndrome (MMIHS) in 1.1% or Cloacal Malformations in 0.7% of cases. Cloacal malformations usually affect female fetuses and carry an extremely bad prognosis. In one third of all published series a definite diagnosis of the underlying pathology was not made [2, 4–7, 16, 17, 31, 40, 51, 52]. Our cohort showed comparable results concerning type and prevalence of the underlying pathology, with PUV or stenotic or atretic urethra in 46%, 20% and 15% of cases, respectively. Neurogenic bladder due to MMIHS or Prune belly syndrome occurred in 5% each, and primary VUR due to insufficient flap-valves in 2.6%. Indeed, in 7.7% of our cases no underlying obstructive or non-obstructive pathology for megacystis could be detected postnatally, although all newborn babies had significant additional urinary anomalies including megaureters, urinoma or end-stage cystic–dysplastic kidney failure. These findings suggest that there must have been a disturbed urinary bladder drainage in the past maybe due to a stenotic or hypoplastic urethra that spontaneously improved through the general growth in size during ongoing pregnancy. Until now, no accurate sonographic marker was identified to reliably differentiate the underlying pathology in the prenatal situation. Therefore, the precise diagnosis of the underlying pathology still remains uncertain until birth and parents should be made aware of the residual risk of long-term urinary, intestinal, neurological and motoric co-morbidity in cases of complex megacystis or cloacal malformations. All physicians involved in the prenatal situation should be aware that due to the prenatal intervention a subset of severely affected children survive that would have died without intrauterine treatment. Especially affected female patients with complex cloacal malformations might not be corrected satisfactorily in some cases, leading to severe livelong morbidity and impairment. Accordingly, among the 3 female babies of our cohort with postnatally confirmed cloacal malformation the diagnosis was prenatally suspected in only 2 cases. In the third case, pregnancy was terminated prior to 22 weeks of gestation when intestinal and genital anomalies cannot be detected yet. Therefore, definite diagnosis was achieved postmortem.

Previously published studies described a low sensitivity of the ‘keyhole sign’ in detecting PUV [43, 53], as it was also observed in some fetuses with PBS and MMIHS [54]. Therefore, prenatal diagnosis of PUV was erroneous in more than 50% [1, 2, 54]. We could confirm that the ‘keyhole sign’ is no reliable marker to predict the underlying pathology or to differentiate between obstructive or non-obstructive megacystis (*p* = 0.137). However, we could demonstrate that the presence of the ‘keyhole sign’ was a reliable predictor of isolated megacystis (*p* = 0.001) even in the first trimester. In contrast, female sex was associated with complex megacystis in all cases and overall survival in female fetuses (with or without ‘keyhole sign’) was only 20%. We concluded, that the ‘keyhole sign’ is a reliable predictor of good outcome, whereas the absence of the ‘keyhole sign’ and female sex predict high mortality and morbidity. Those prognostic markers might help counseling parents in the prenatal situation.

In our cohort, 8 fetuses underwent late VAS > 17 + 0 weeks, all had severe oligo- or anhydramnios. First VAS in this subset of fetuses was performed at an average of 22 weeks of gestation (range, 19–26 weeks). Although some reports suggested that megacystis first diagnosed in the second trimester is more likely to result in live births [5], oligo- or anhydramnios was reported to be a strong predictor of lung hypoplasia and neonatal death [40, 42]. Therefore, we decided to perform late VAS, and although shunt complications occurred in 75% of cases and renal function was impaired in 37.5% postnatally, all fetuses survived with adequate pulmonary size and function without any need for mechanical respiratory support. We conclude that late VAS may be too late to prevent kidney damage in all cases but it may have a positive impact on pulmonary development and survival.

Fetal urine production starts as early as 9 weeks of gestation and the urinary bladder can be identified sonographically from this week on. In addition, nephrogenesis begins early at 4 weeks of gestation and is not completed prior to 34–36 weeks of gestation [47]. Because impaired drainage due to early onset obstruction of the lower urinary tract carries a substantial risk of impaired glomerulo- and nephrogenesis and longer duration significantly worsens kidney function, early detection of megacystis with timely intervention is crucial to avoid irreversible kidney damage. Therefore, every sonographer performing screening ultrasound in pregnancy should be aware of early signs of impaired bladder drainage and megacystis, especially during first trimester examinations.

A limitation of this study is its retrospective design. Furthermore, our cohort is small due to the naturally low prevalence of megacystis. In addition, although our trial described one of the largest single-center series so far, the total number of treated fetuses is still small and larger prospective series are desirable in future. A standardized approach to timing the VAS is needed. Therefore, we started a prospective clinical trial on VAS in the first trimester using the Somatex shunt with standardized postnatal follow-up protocol in our center in 2021 (DRKS00017779). In addition, a re-evaluation of the cohort described in this present paper will be conducted next year to assess the mid- and long-term effects of VAS on kidney and pulmonary function.

## Conclusions

Early prenatal VAS is feasible from 12 weeks onward, can preserve neonatal kidney function in the majority of cases and enables survival in up to 96% of cases. It should be no longer a matter of debate that VAS should be offered to all fetuses with moderate or severe megacystis as early as possible. However, despite successful fetal intervention, parents should be aware of the potential risk of postnatal mid- or long-term kidney failure and of additional health impairment due to concomitant extra-renal anomalies that cannot be excluded at time of intervention.

## Data Availability

The author (IG) can confirm that all relevant data are included in the article.
